# Standardized collection of MNase-seq experiments enables unbiased dataset comparisons

**DOI:** 10.1186/1471-2199-13-15

**Published:** 2012-05-06

**Authors:** Jason M Rizzo, Jonathan E Bard, Michael J Buck

**Affiliations:** 1 Department of Biochemistry, State University of New York, Buffalo, NY, USA; 2 Center of Excellence in Bioinformatics and Life Sciences, State University of New York, Buffalo, NY, USA; 3 Genetics, Roswell Park Cancer Institute, Buffalo, NY, USA

**Keywords:** Next-generation sequencing, High-throughput sequencing, Chromatin, Nucleosomes, Histones, MNase-seq, Micrococcal nuclease (MNase)

## Abstract

**Background:**

The organization of eukaryotic DNA into chromatin has a strong influence on the accessibility and regulation of genetic information. The locations and occupancies of a principle component of chromatin, nucleosomes, are typically assayed through use of enzymatic digestion with micrococcal nuclease (MNase). MNase is an endo-exo nuclease that preferentially digests naked DNA and the DNA in linkers between nucleosomes, thus enriching for nucleosome-associated DNA. To determine nucleosome organization genome-wide, DNA remaining from MNase digestion is sequenced using high-throughput sequencing technologies (MNase-seq). Unfortunately, the results of MNase-seq can vary dramatically due to technical differences and this confounds comparisons between MNase-seq experiments, such as examining condition-dependent chromatin organizations.

**Results:**

In this study we use MNase digestion simulations to demonstrate how MNase-seq signals can vary for different nucleosome configuration when experiments are performed with different extents of MNase digestion. Signal variation in these simulations reveals an important DNA sampling bias that results from a neighborhood effect of MNase digestion techniques. The presence of this neighborhood effect ultimately confounds comparisons between different MNase-seq experiments. To address this issue we present a standardized chromatin preparation which controls for technical variance between MNase-based chromatin preparations and enables the collection of similarly sampled (matched) chromatin populations. Standardized preparation of chromatin includes a normalization step for DNA input into MNase digestions and close matching of the extent of digestion between each chromatin preparation using gel densitometry analysis. The protocol also includes directions for successful pairing with multiplex sequencing reactions.

**Conclusions:**

We validated our method by comparing the experiment-to-experiment variation between biological replicates of chromatin preparations from *S. cerevisiae*. Results from our matched preparation consistently produced MNase-seq datasets that were more closely correlated than other unstandardized approaches. Additionally, we validated the ability of our approach at enabling accurate downstream comparisons of chromatin structures, by comparing the specificity of detecting Tup1-dependent chromatin remodeling events in comparisons between matched and un-matched wild-type and *tup1*Δ MNase-seq datasets. Our matched MNase-seq datasets demonstrated a significant reduction in non-specific (technical) differences between experiments and were able to maximize the detection of biologically-relevant (Tup1-dependent) changes in chromatin structure.

## Background

DNA-histone interactions are the first order of chromatin structure, and both the strength and locations of these interactions have significant influence on the accessibility and regulation of genetic information [1,2]. DNA-histone interactions have been characterized in a variety of model organisms on both genome-wide and site-specific scales using various methods [3-6]. Genomic studies of chromatin structure most often focus on key structural relationships shared by cell populations, including the density of nucleosomes at a given DNA locus (known as ‘nucleosome occupancy’) and the extent to which nucleosomes resist deviating from consensus locations along DNA (known as ‘nucleosome positioning’)[7].

Technical approaches to the measurement of chromatin structure generally consist of two phases: collection of DNA associated with a particular type of chromatin and characterization of the isolated nucleic acid pool [8]. Collection of chromatin-associated DNA typically solicits the use of micrococcal nuclease (MNase), an endo-exo nuclease that preferentially digests naked DNA and enriches for nucleosome-associated DNA (nucleosomal DNA) [5,9,10]. Nucleosomal DNA can be sequenced using high-throughput DNA sequencing (MNase-seq) to provide a genome-wide view of chromatin structure [3,5,11,12]. MNase-seq experiments have generated genome-wide maps of chromatin in both humans and model organisms [13,14]. The *Saccharomyces cerevisiae* (baker’s yeast) model was the first organism to have its chromatin mapped using MNase-seq, and it remains the most extensively studied model with dozens of MNase-seq datasets available today.

DNA signals gathered from MNase-seq experiments are believed to reflect protection from MNase digestion (MNase protection) and to subsequently relate to underlying nucleosome occupancies. Despite these common assumptions, however, MNase-seq signals have shown considerable variation between technical preparations, especially at nucleosomes surrounding transcription start sites (TSSs). For example, recent work in *S. cerevisiae* by Weiner *et al.* and Xi *et al.* has demonstrated the presence of easily digested nucleosomes and/or other protein complexes present in the chromatin pool of under-digested nuclease preparations that were absent in preparations using greater digestion levels[15,16]. Additionally, work by Kent *et al.,* also in *S. cerevisiae,* has demonstrated the preferential enrichment of small MNase-protected regions in incompletely digested chromatin samples which map to nucleosome-depleted regions and are not present in other chromatin preparations in the literature [17].

Ultimately, the variable nature of MNase-seq signals suggests technical variance in chromatin DNA sampling and is a confounding factor when drawing comparisons between different MNase-seq experiments. Accordingly, many chromatin researchers have noted this influence and advocate the comparison of only similarly prepared datasets to limit the influence of technical differences [18,19]. Despite these suggestions, no protocol exists to guide the collection of such data. Therefore, in this study we present a standardized method for the collection of matched MNase-digested samples which reproducibly sample the same DNA populations and are therefore comparable. We validate the specificity of our approach by comparing our matched samples with unmatched samples prepared in our own lab and by other groups. Additionally, we also outline the best approaches for analyzing these datasets to enhance downstream comparisons.

## Results

### DNA sampling differences exist between distinct MNase-seq preparations

**Figure 1:**
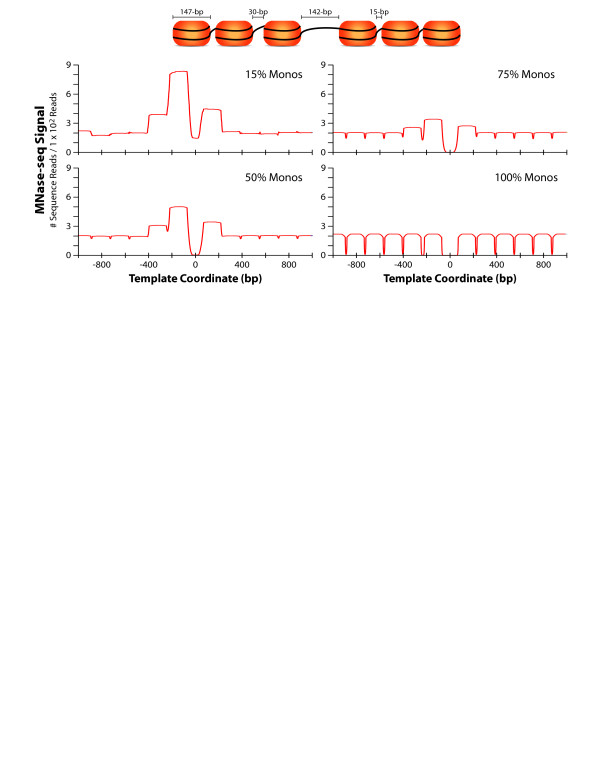
**Sampling of different nucleosome configurations changes with extent of MNase digestion**. Computer simulation of an MNase digestion titration depicting different technical preparations of chromatin including under-digested samples (15% and 50% Monos), normal-digested samples (75% Monos), and complete-digested samples (100% Monos). Graphs illustrate the normalized count (# sequence reads) of mono-nucleosome fragments aligned to the original template sequence after a simulated digestion and size selection (115–195 bp). All simulations were conducted on an identical template population with each template containing 6 equally sized nucleosomes (147-bp) with 100% occupancy. Each nucleosome was spaced 15-bp apart except for a central 142-bp linker and a larger upstream linker (30-bp). Nucleosomal protection of DNA is modeled to range from 50x to 1000x greater than naked (linker) DNA and relates to a base-pair’s location within a nucleosome, based on the *in vivo* work of Widom and colleagues (see Additional file 2: Figure S5) [20].

Protocols for the collection of chromatin DNA utilize gel electrophoresis to isolate and select mono-nucleosome DNA following MNase digestion of a chromatin population. This isolated DNA is assumed to accurately represent a fair sampling of nucleosomes, and corresponding chromatin structures, in the initial population at every position in the genome. This assumption allows researchers to make relevant comparisons between different genomic locations within a sample and between samples. However, for most MNase experiments this assumption is invalid, because some nucleosomes are easier to excise from chromatin than others and therefore their DNA is more likely to be sampled and sequenced in MNase-seq experiments. The simulation in Figure 1 illustrates this principle, showing how the same, equally occupied, chromatin template can yield a range of MNase-seq signals due to sampling differences between distinct nucleosome configurations across different extents of MNase digestion (% Monos). In these simulations, nucleosome configurations with or near to longer neighboring linker DNA lengths were sampled differently, depending on the extent of MNase digestion. Sampling differences in Figure 1 were more pronounced at lower extents of MNase digestion (15% and 51% Monos), however, this sampling bias was also seen for a simulation of a typical (normal) MNase digestion preparation (76% Monos), indicating that sampling bias can contribute to MNase-seq signals in typical chromatin preparations. Differences in nucleosomal DNA sampling represent a neighborhood effect of MNase digestion techniques. Size-selection of mono-nucleosomal DNAs following MNase digestion makes the representation of each individual nucleosomal DNA sequence in that population dependent on having two MNase cuts both up and downstream of that location and within a size range captured by gel excision (~115-195-bp). Ultimately, the likelihood of achieving these two cuts at the appropriate locations increases significantly when a nucleosome’s location is flanked by longer stretches of linker (unprotected) DNA. This result is due to the dramatic difference in linker DNA digestion rates compared to those of nucleosomal DNA, which ranges from 50X-1000X difference or greater *in vivo* depending on a base-pair’s location within a nucleosome[20].

**Figure 2:**
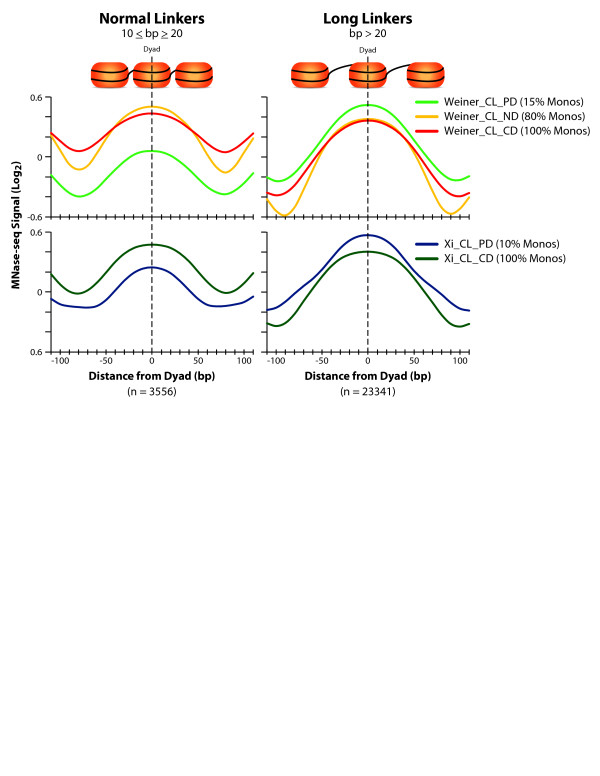
**Neighboring linkers drive MNase-seq signal measurements**. Graphs illustrating the relative MNase protection signals at specific nucleosome configurations *in vivo* with either normal or long neighboring linker DNA. Linker lengths and nucleosome positions were defined by Jiang *et al.*[32]. *TOP:* MNase protection data from a partial digest (PD; *light green*), normal digest (ND; *yellow*), and from a complete digest (CD; *red*) from Weiner *et al.*[15]. *Bottom:* MNase protection data from partial digest (PD; *blue*) and a complete digest (CD; *dark green*) from Xi *et al.*[16].

A similar result can be seen *in vivo* when comparing the MNase-seq signals collected for two distinct nucleosome configurations across different technical preparations (Figure 2). Nucleosomes with longer neighboring linker DNAs show increased signal relative to mono-nucleosomes with normal linkers at lower extents of MNase digestion (10% or 15% Monos), and this over-representation decays as the extent of digestion increases (80% or 100% Monos). Conversely, nucleosomes with normal linker sizes show the opposite result, with decreased signal relative to long-linker mono-nucleosome populations at lower extents of MNase digestion and increased signal with increased extents of digestion. Importantly, these results provide actual (not simulated) examples of how MNase-seq signal changes correlate with neighboring linker lengths.

### Standardized collection of matched chromatin samples

Together, results in Figures 1&2 demonstrate how differences in nucleosomal DNA sampling across different chromatin preparations for the same template can alter MNase-seq signals. Ultimately, this variation will confound comparisons between different MNase-seq experiments. Therefore, we developed a method to minimize technical differences between chromatin preparations, matching the extent of MNase digestion, completely digesting chromatin, and removing extra size-selection steps (gel excision) to collect comparable MNase-seq experiments. Differences in representation of nucleosomal DNAs between these comparable or “matched” datasets relate to true biological differences in chromatin structure, since comparisons are no longer confounded by technical artifacts introduced by sampling differences.

#### Overview

**Figure 3:**
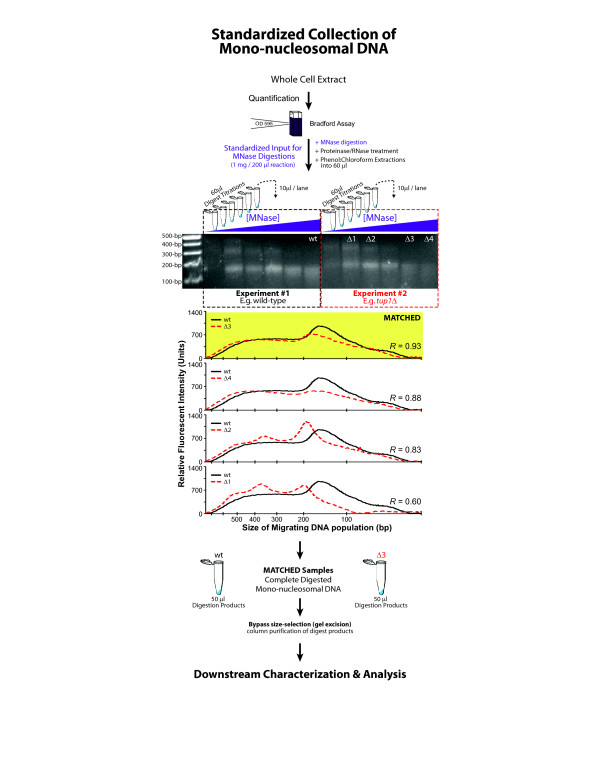
**Flow-diagram of standardized mono-nucleosomal DNA collection**. A standardized amount of whole-cell extract (WCE) is used as input for MNase digestions to isolate nucleosomal DNA and collect mono-nucleosomal fragments. A small fraction of nucleosomal DNA then is separated following MNase digestion using gel electrophoresis. The extent of MNase digestion is compared between these samples by calculating a Pearson correlation (*r*) for relative front (i.e. separation relative to standard fragment sizes) for the migrating chromatin DNA populations in each lane. ‘Matched’ digests are selected as two complete-digested (100% Monos) samples that have a correlation coefficient r > 0.9 (*yellow* highlight). Remaining nucleosomal DNA (not loaded onto the gel) is then column purified and used in downstream analysis, thus bypassing the need for gel-excision.

The general work-flow for chromatin DNA preparations begins by collecting a cell population whose chromatin structure is fixed. Fixation is achieved *in vivo* by chemical cross-linking with formaldehyde treatment and is necessary to prevent histone exchange during chromatin purification[21]. Fixed chromatin is assayed through extracting and isolating DNA specifically associated with nucleosomes, using a combination of cell/nuclear lysis (chromatin extraction), MNase digestion (nucleosomal DNA isolation), and electrophoretic separation (mono-nucleosomal DNA purification) methods. To reduce variance in this DNA preparation, we have standardized the extraction, isolation, and collection of nucleosomal DNA as described below (Figure 3 and Additional file 1: Supplemental Protocol). Our protocol standardization was successful at reducing technical variation between MNase chromatin preparations and allowed for highly reproducible results.

#### Chromatin extraction

Chromatin extraction will depend on the specific cell type and organism. For most samples chromatin extraction can be performed in the same manner as employed by chromatin immunoprecipitation (ChIP) experiments as long as the samples are cross-linked (formaldehyde fixed). For *Saccharomyces cerevisiae*, we found that the conventional approach of isolating spheroplasts for MNase assays was generating considerable technical noise. Therefore, to eliminate variance associated with spheroplasting we instead extracted intact chromatin as part of a whole-cell extract (WCE) using mechanical disruption of cell walls and nuclei (bead-beating). This approach is the same as applied to most yeast ChIP protocols and is compatible with other cell types including human cells [22,23]. WCEs provided a consistent and reproducible yield of chromatin input for downstream nucleosomal DNA preparations steps. WCEs were stored at −80°C in small aliquots, each providing input for several MNase reactions and reducing the need for freeze-thawing samples (see Additional file 1: Supplemental Protocol).

#### Nucleosomal DNA isolation

We utilized a Bradford assay to quantify the total protein yield of our WCEs and standardize the amount of the WCE (and corresponding input DNA) going into each downstream MNase digestion reaction. Standardizing DNA input for MNase digests ensures reproducibility of digest results. Additionally, Bradford readings also provided a quality control to ensure chromatin extraction techniques were successful at lysing cells and nuclei, since low/inconsistent readings would warn against uneven chromatin yields between experiments. Alternatively, a DNA-based quantification (Hoechst) assay can be used in place of the Bradford Assay for quantification and standardization measures.

A range (titration) of MNase digestions was performed to isolate nucleosomal DNA and to identify the extent of digestion desired (Figure 3). MNase digestions are performed using longer digestion times (1 hour at 37 C) and lower concentrations of MNase to reduce the time-dependence of results from this step. Additional digestion series can be employed as needed to ensure collection of several similarly digested (matched) samples at the desired extent of digestion (see: Identifying Matched MNase digests).

#### Mono-nucleosomal DNA purification

Most investigators choose to collect mono-nucleosomal DNA from a moderately digested chromatin population (~80% mono-nucleosomes)[15]. While this is possible with our protocol, we believe that collection of more completely digested chromatin samples (~95-100% monos) enables greater reproducibility. Specifically, since completely digested chromatin is mostly composed of mono-nucleosomal DNA, our approach removes the need for an extra size selection to collect mono-nucleosomal DNA before generating the Illumina sequencing libraries which is another potential source of technical variation[12,24].

Mono-nucleosomal DNA remaining from complete digests (i.e. non-gel-purified) is collected and purified following standard chromatin preparation procedures, including crosslink reversal, proteinase-K digestion, a series of phenol:chloroform extractions, and RNase treatment. The purified mono-nucleosomal DNA is subsequently analyzed to select matched samples for experimental characterization (see Additional file 1: Supplemental Protocol).

#### Identifying matched MNase digests

Matched digests were collected by characterizing a small quantity (10 μl) of standardized MNase digestion products using ethidium bromide staining and gel densitometry analysis. The practical limit of detection with this approach is 5 ng/single band of DNA with a dynamic range of 5 ng to greater than 1 μg depending on fluorescent exposure times[25]. Briefly, gel intensity measurements for each lane were calculated using standard densitometry software provided by Biorad (Quantity One^TM^) and exported to Microsoft Excel^TM^ for correlation analysis (Pearson). A 100 bp standard ladder (Qiagen) was analyzed on the same gel to calculate a relative front (i.e. separation relative to standard fragment sizes) for the migrating DNA population. Correlation coefficients between relative front distributions were calculated for digest titration levels no longer showing a visible di-nucleosome band (i.e. completely digested samples) and spanning a region known to cover a size range of 0–400 bp of DNA (Figure 3). ‘Matched’ digests were selected as two completely digested samples having a correlation coefficient (*r*) > 0.9. Correlation coefficients *r >* 0.9 were selected because these showed the most reproducible change in MNase protection data on single site real-time quantitative PCR (qPCR) analysis (Additional file 2: Figure S1). The remaining sample from each digest was column-purified (Zymo Research), bypassing the need to gel excise mono-nucleosome DNA.

#### Sequence library preparation

Sequencing libraries should be prepared following standard ChIP-seq protocols with the following adjustments: Depending on your DNA recovery it is likely that you will recover more mono-nucleosome DNA than what is standard for a ChIP-seq protocol. The standard ChIP-seq protocol is optimized to 10 ng of total DNA and should be adjusted at the adaptor ligation step according to the amount of recovered DNA. Additionally, when cleaning up your adaptor-ligated library on an agarose gel, care should be taken to ensure that the gel excisions are the same size and in the same range to avoid generation of added technical noise.

We have also successfully paired our protocol with Illumina’s Low Throughput TruSeq library preparation. This protocol allows for a gel-free method of library preparation and also enables multiplexing of sequencing reactions. Sequencing libraries should be prepared following this method with the following exceptions: use only 200 ng of mono-nucleosomal DNA as input (quantified by picogreen assay[25]) and replace the first AmpPure bead-cleanup step (after End Repair and before A-Tailing reactions) with a MinElute purification step (elute with 30 μl elution buffer). This step removes the size-selection bias of bead-cleanups, which favors larger (x > 200 bp) DNA fragments. After the column cleanup and 3’ adenylation, use 1:5 diluted adapters for ligation reactions and proceed as directed. Finally, when enriching DNA fragments, follow the listed PCR conditions, but only use 1μl of ligation products as input to ensure the PCR primers remain in excess and to avoid bubble amplification products. This protocol allows up to 24 samples to be multiplexed into a single sequencing lane.

### Standardized analysis of MNase-seq data

Genomic approaches to mapping chromatin present challenges in downstream analysis, specifically in converting large amounts of short-read DNA sequences into biologically-relevant information about chromatin structures. Most importantly, accurate analysis of and comparisons between MNase-seq experiments require these datasets to be processed identically, since subtle differences in processing can alter signals and ultimately confound downstream comparisons. We provide an outline of several approaches for the analysis and comparison of MNase-seq datasets, since a consensus method has not been established (See Additional file 3: Supplemental Methods).

### Validation of method: Matched MNase preparations reduce variation between biological replicates of MNase-seq

**Figure 4:**
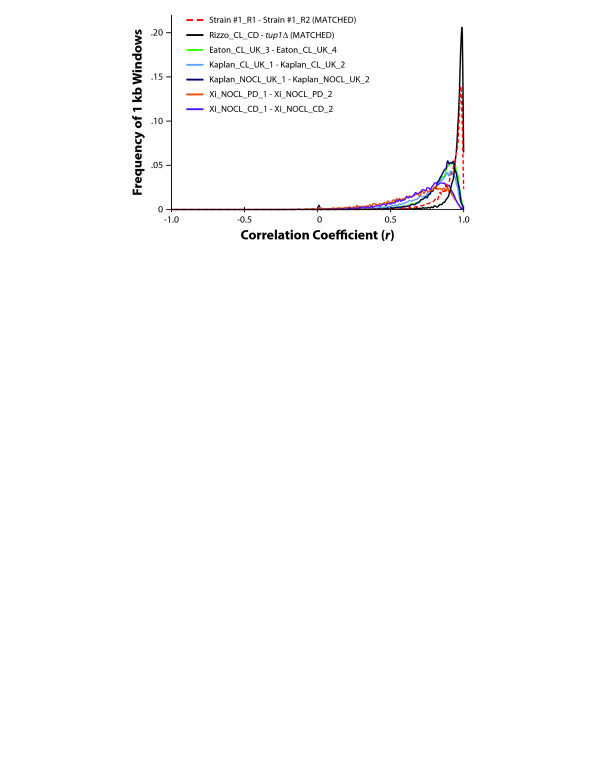
**Matched samples yield fewer dissimilar regions of chromatin**. Histogram illustrating the frequency of Pearson correlations (*r*) for comparisons between MNase-seq datasets for all tiling 1000 bp windows across the *S. cerevisiae* genome at 10 bp resolution. Comparisons were made between biological replicates, including our matched digest experiment (Strain #1 R1 & R2; *red dotted line*) and other published wild-type replicates (see Additional file 2: Table S1 for experimental details and references). Additionally, a comparison of matched wild-type and *tup1*Δ MNase-seq data is also illustrated (*black*). Results demonstrate how our matched preparation consistently produces MNase-seq datasets that are more closely correlated than other unstandardized preparations.

We validated our method by comparing the experiment-to-experiment variation between biological replicates of chromatin preparations from *S. cerevisiae*. A Pearson correlation coefficient was calculated comparing standardized MNase-seq data between biological replicates for 1000 bp windows tiling across the yeast genome. In these experiment-to-experiment replicate comparisons, technical differences in collection of mono-nucleosome DNA populations will appear as dissimilar (poorly correlated) chromatin regions. A distribution of our correlation analysis is illustrated in the histogram in Figure 4. Results from our matched preparation consistently produced MNase-seq datasets that were more closely correlated than other unstandardized preparations. For our matched chromatin samples (*red dotted line*), 69% of all 1000 bp windows demonstrated an *r* > 0.9 with less than 1% having *r* < 0.5. Additionally, this figure also illustrates how matching chromatin samples for two distinct chromatin structures (wild-type and *tup1*Δ; *black*) also reduces technical variation between preparations to less than that seen between two biological replicates of the *same* strain done by various groups. This ability to prepare closely matched samples in distinct genome strains or conditions enables unbiased comparisons of MNase-seq datasets (see next section).

### Validation of method: Matched MNase preparations detect specific differences in chromatin structures

To validate the specificity of our matched MNase-seq protocol we compared our matched wild-type and a *tup1*Δ MNase-seq datasets with relation to Tup1p binding. Since Tup1p is known to stabilize nucleosome positioning and occupancy directly *in vivo*, biologically-relevant differences between wild-type and *tup1*Δ chromatin structures should map close to Tup1-binding sites [19,26-29]. Dissimilar chromatin structures between datasets were identified as described previously using a sliding Pearson correlation (bp ≥ 1000, *r* < 0.5) and the average Tup1 binding for these locations was calculated to evaluate the specificity of chromatin structure differences to Tup1 function [19]. As illustrated in Additional file 2: Figure S2, our matched dataset (*yellow highlight*) demonstrated a marked enrichment of Tup1 binding at dissimilar chromatin structures when comparing wild-type and *tup1*Δ datasets. Moreover, when compared to a random sampling of 10,000 chromatin regions, the enrichment of Tup1 binding at these regions was shown to be statistically significant (*p* = 3.75 × 10^-35^). The ability to detect Tup1 enrichment decreased when an unmatched wild-type MNase-seq dataset was used for the comparison. This result was due to the identification of non-specific changes in chromatin structures introduced by technical differences and occurred regardless of the dissimilarity metric we utilized in dataset comparisons (Additional file 2: Figure S2–S4).

## Discussion

Recent work has called attention to how MNase-seq signals change across different preparations of chromatin, specifically those with dramatically different extents of MNase digestion [15-17]. While several groups have mined these changes to identify potentially biologically relevant phenomena, our computer simulations illustrate an important DNA sampling bias present in MNase-seq experiments which can also alter MNase-seq signals between different preparations of chromatin (Figure 1). Moreover, results in Figure 2 provide experimental data that highlight how changes in MNase-seq signals between different chromatin preparations correlate with the DNA sampling bias outlined by our computer simulations. While characteristics of nucleosomal DNAs may also be contributing to the MNase-seq signals seen in Figure 2 and published elsewhere, the neighborhood effect of MNase digestion techniques is also contributing to signal variation and thus confounds comparisons between MNase-seq datasets with different digestion conditions.

Our standardized approach to MNase-seq experiments was designed to enable the collection of similarly sampled chromatin populations which minimizes the influence MNase sampling biases when matched MNase-seq experiments are compared, such as the chromatin from wild-type and *tup1*Δ yeasts (Additional file 2: Figure S2). While matching any extent of MNase digestion (i.e. partial or complete) ensures this control for MNase-seq dataset comparisons, we feel that more reproducible data can be generated using complete digested chromatin given the ability to bypass additional size-selection steps. Additionally, we favor complete digested chromatin because the MNase-seq signal in these preparations is no longer driven by nucleosome configuration. This is because complete digestion (100% Monos) requires all linker regions to have been cut at least once, thus minimizing sampling bias (Figure 1).

## Methods

### MNase-seq datasets

Biological replicates for Strain #1(Genotype: MATa,ade2-101(*ochre*), his3-Δ200, leu2-3,112, lys2-801(*amber*),trp1-Δ901,ura3-52, GAL+,thr-,tyr-,arg4-1, Δh4-1,[HIS3+], Δh4-2[LEU2+]/pUK499(URA3+,H4-2+)) were prepared using our standardized protocol (matched digestions) including cross-linking and complete digestion of chromatin samples. Cells were fixed during log phase and asynchronized growth in rich media with galactose as the carbon source (YPG). All other MNase-seq datasets were downloaded from SRA and processed identically [30]. Additional file 2: Table S1 lists the SRA accession numbers, experimental parameters, and unique identifiers for all of the MNase-seq datasets utilized in this study. All MNase-seq data were processed identically, including alignment to the most recent genome build available on the *Saccharomyces cerevisiae* Genome Database (SGD build r64; www.yeastgenome.org) using the Bowtie alignment algorithm allowing only unique matches with up to 2 mismatches. [31]. Genome-to-genome Pearson correlations between wild-type and *tup1*Δ datasets were calculated at 100-bp resolution in Microsoft Excel^TM^.

### MNase digestion simulations

Digest simulations utilized a 4 kb template with nucleosome (147 bp) and linker (15 bp) DNAs sized according to the average sizes of a *S. cerevisiae* reference nucleosome atlas defined by Jiang *et al.*[32]. Additionally, the simulation template also included a 142-bp central linker and the larger upstream linker (30 bp) to represent the average nucleosome organization seen upstream of TSSs in *S. cerevisiae*[19,32]. Simulations assumed equal kinetics for digestion of all nucleosomal DNAs, similar to assumptions of other rate-dependent chromatin analysis [33]. MNase cuts were randomly distributed to the DNA sequences on each template and made based on a probability function whereby linker DNAs were always cut and nucleosomal DNAs were scaled in protection according to a base-pair’s location within a nucleosome (Additional file 2: Figure S5). This randomized simulation mimics the average distribution of MNase cuts in a given digest. Simulations were conducted using a range of cut numbers (n = 2–50) to mimic different extents of digestion. Each extent of digestion (i.e. (n) # cuts) was then iterated for 10,000 templates to simulate digestion of a chromatin template population.

Following randomized cut distributions (i.e. simulated digestion), potential mono-nucleosomal DNA fragments (sized 115 < bp >195) were then size-selected from the remaining fragment sizes in the entire chromatin template population and aligned to the template sequence for signal tabulation. This assumes that the entire length of each fragments was sequenced, the functional equivalent to a paired-end run, and bypasses the need for sequence tag extension which would introduce additional and unnecessary assumptions into our simulations. The count of aligned mono-nucleosome fragments was standardized between simulations to control for differences in fragment numbers between experiments (similar to standardizing the number of sequence reads in MNase-seq experiments described in Additional file 3: Supplemental Methods). To eliminate the edge effect at the ends of 4 kb templates when viewing aligned and standardized mono-nucleosomal DNA fragment signals, only a central 1000 bp window of each template was plotted (Figure 1) and data was normalized to the signal at template coordinate 2500 bp-3500 bp, which was identical in all simulations. The extent of MNase digestion was determined by counting the total number of mono-nucleosomal DNA fragments divided by the total number of remaining DNA fragments (size >115 bp) to estimate the extent of MNase digestion (% Monos).

### Reference nucleosome comparisons

Nucleosome configurations in Figure 2 were identified from a reference dataset of *S. cerevisiae* nucleosome positions defined by Jiang *et al.*[32]. Reference nucleosome positions were lifted over to the r64 build of the yeast genome utilized in this analysis using the LiftOver program on SGD. Adjacent linker sizes (left and right) were calculated for all reference nucleosomes. Normal linkers were defined to include a size range centered on the mean linker size identified by Jiang *et al.* (10 ≤ bp ≥ 20) and long linker sizes were defined as exceeding this range and extending to include sizes ~1.5X the size of an average nucleosome template (21 < bp > 221). The averaged MNase protection profiles in Figure 2 were plotted for nucleosomes with both adjacent linkers of the same size type (normal or long).

### Analysis of dissimilar chromatin regions

Tup1 binding data overlapping dissimilar chromatin regions was taken from ChIP-chip experiments by *Hanlon et al.*[29]. Continuous binding (log_2_ ratio) profiles were generated by ChIPOTle's sliding window approach [34]. Averaged continuous binding data overlapping dissimilar regions was calculated as described previously [19]. Only regions with available ChIP-chip data were included in the analysis. Random windows consisted of 10,000 randomly sampled chromatin regions with sizes equivalent to the mean size for dissimilar windows between matched wild-type and *tup1*Δ chromatin. The statistical significance of these values was tested by comparing the distribution of Tup1-binding values for dissimilar windows against the binding values for 10,000 random regions of chromatin, using a one-tailed heteroscedastic *t*-test.

## Competing interests

None declared.

## Author’s contributions

JMR performed the experiments and analyses. JEB created the MNase digestion simulator used in the analysis. JMR and MJB designed the study and wrote the manuscript. All authors read and approved the final manuscript.

## Supplementary Material

Additional file 1 Supplementary Protocol. Matched Micrococcal Nuclease Digestions. Click here for file

Additional file 2 **Supplemental Figure 1.** Unmatched preparations show poor consistency in identifying changes in MNase protection signals. Supplemental Figure 2. Matched MNase digests identify biologically relevant differences in chromatin structure. Supplemental Figure 3. Comparison between wild-type and tup1? MNase-seq experiments at a single region of Tup1-dependent chromatin. Supplemental Figure 4. The ability of matched MNase digests to specifically detect biologically-relevant differences in chromatin structure is NOT dependent on dissimilarity cutoff values used in analysis. Supplemental Figure 5. MNase cut probability function for nucleosomal DNA templates. Supplemental Table 1: MNase-seq datasets used in this study. Click here for file

Additional file 3 Supplemental Methods: Standardized MNase-seq Analysis. Click here for file
