# Supplementary material for: Standardized collection of MNase-seq experiments enables unbiased dataset comparisons
Source: BMC Mol Biol. 2012 May 6;13:15. doi: 10.1186/1471-2199-13-15 (PMC3464627; doi:10.1186/1471-2199-13-15)
Supplement: Additional file 1 — Supplementary Protocol. Matched Micrococcal Nuclease Digestions. [file 1471-2199-13-15-S1.pdf]

Supplemental Methods

Both MNase-qPCR and histone chromatin immunoprecipitation (ChIP) qPCR for the dissimilar region of chromatin in **Supplemental Figure 1** (iYLR295C) were performed as described previously [1]. Unmatched samples in **Supplemental Figure 1** were collected following the same MNase-digestion protocol as matched samples, but included gel-excision steps and selected only samples with gel smear correlations $r < 0.7$. Additionally, two groupings of unmatched samples were made according to extent of MNase digestion (% Monos) estimated by gel imaging analysis. Unmatched samples 1 represent normal to complete digestion (x > 50% Monos) and Unmatched samples 2 represent under-digestion (x < 50% Monos). These groupings were made to avoid averaging out sampling differences in unmatched signal comparisons.

Supplemental Validation of Method:

To further examine our matched datasets we also compared our genome-wide *tup1*Δ chromatin data to multiple wild-type chromatin datasets. Since we could not calculate gel-front correlations between our data and other MNase-seq experiments, we determined a genome-to-genome Pearson correlation between datasets to gauge how well-matched they are to our tup1D data (**Supplemental Table 1**).

As evident by the trend in **Supplemental Figure 2**, Tup1-specific differences in chromatin structure between wild-type and *tup1*Δ chromatin were seen for only highly-correlated datasets (*orange and pink highlights*), and our matched sample (*yellow highlight*) preparation provided the greatest Tup1-enrichment score. Importantly, the loss of Tup1-enrichment signal seen for dissimilar chromatin regions in unmatched sample comparisons was due to identification of non-specific changes in chromatin structures caused by technical differences. **Supplemental Figure 3** illustrates this concept, depicting a single dissimilar region of chromatin identified between our matched samples that is centered on a Tup1 binding site. Decreased specificity for unmatched comparisons is observed regardless of the stringency of defining dissimilar regions **(Supplemental Figure 4**).

Overall, these results demonstrate the superiority of our matched method of uniquely identifying Tup1-specific regions of dissimilar chromatin, whereas unmatched datasets suffer from non-specific differences in chromatin structure for all MNase-seq data comparison windows.

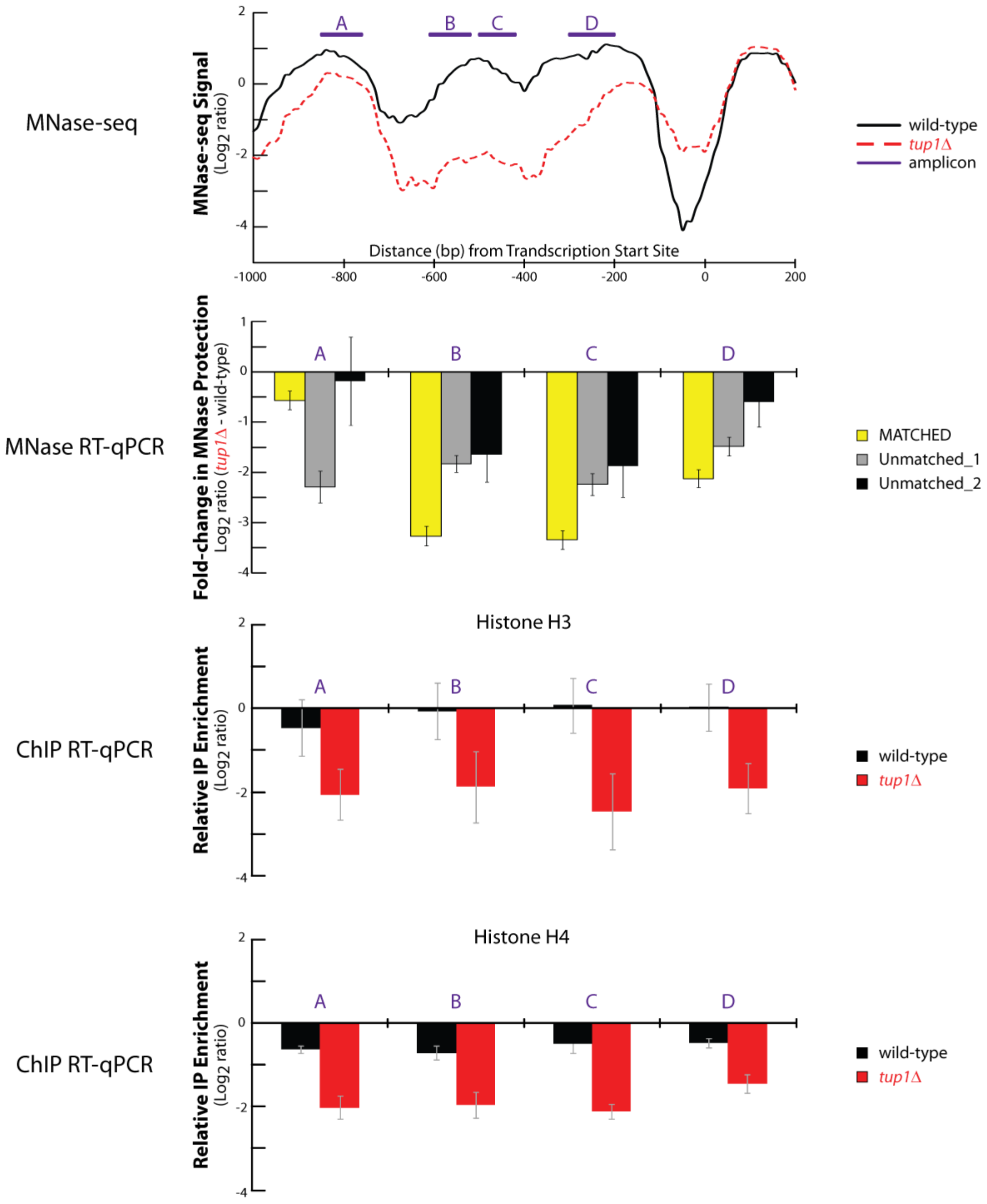

Supplemental Figure 1. Unmatched preparations show poor consistency in identifying changes in MNase protection signals

Graphs illustrating MNase protection data and the underlying nucleosome occupancy data for both wild-type (*black*) and *tup1*Δ (*red*) chromatin. Error bars represent the standard error between experiments.

Top panel: Graph of MNase-seq data for matched samples of wild-type and *tup1*Δ chromatin surrounding the YLR295C transcription start site. MNase-seq data from matched samples illustrates a decrease in MNase protection upstream of the YLR295C gene associated with loss of Tup1-dependent nucleosome stabilization, which has been demonstrated previously [1].

Upper middle panel: Bar graphs illustrating the reproducibility of MNase-seq results using MNase-qPCR approaches on both matched and unmatched chromatin preparations. Matched preparations (*yellow*) of chromatin DNA consistently identify changes in MNase protection signals which relate to the initial MNase-seq signal. These changes also relate to underlying changes in nucleosome occupancy (*lower middle* and *bottom panels*). Unmatched preparations (*black* and *grey*) show poorer ability to identify changes in MNase-seq signals, especially at locations with subtle changes in protection such as amplicons A and D (*purple*). Unmatched preparations also show more variance between preparations (larger standard error).

Lower middle and bottom panels: Bar graphs illustrating the underlying nucleosome occupancy (histone ChIP-qPCR) corresponding to MNase-seq signals upstream of the YLR295C gene.

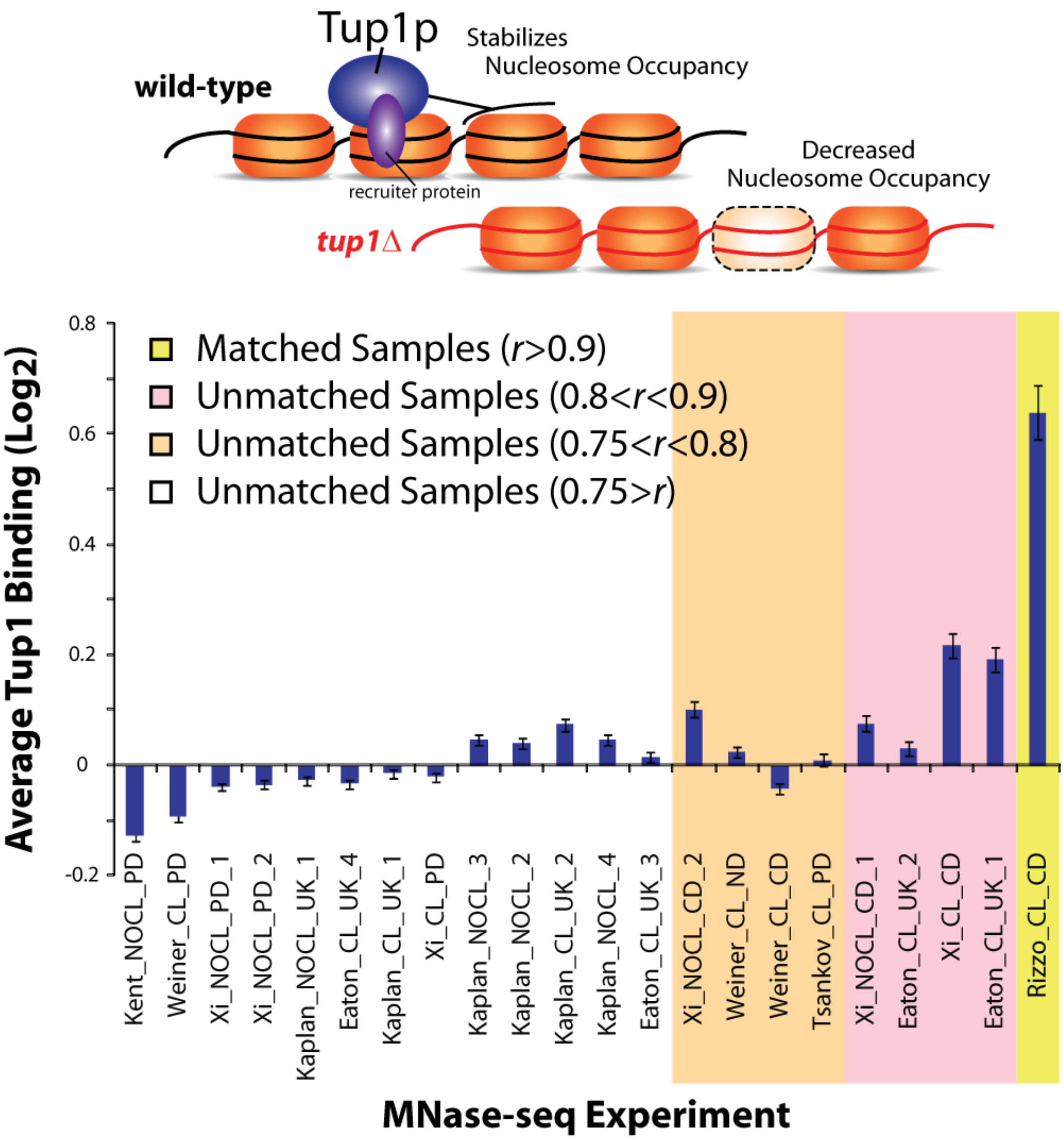

Supplemental Figure 2. Matched MNase digests identify biologically relevant differences in chromatin structure.

Bar graph illustrating the average Tup1 binding (*blue)*, from ChIP-chip analysis [2], for dissimilar chromatin structures identified when comparing various wild-type MNase-seq datasets to our *tup1*Δ dataset. Sources and characteristics of wild-type datasets are listed in **Supplemental Table 1**. All dataset comparisons are sorted by genome-to-genome Pearson correlations between processed wild-type and *tup1*Δ MNase-seq datasets, whereby correlation (*r*) increases along the x-axis reading left to right, such that Kent_NOCL_PD is the lowest correlated dataset and Rizzo_CL_CD is the highest correlated. Tup1-specific differences in chromatin structure between wild-type and *tup1*Δ chromatin are seen for only highly correlated datasets (*pink* and *orange* highlights) and are best captured by our matched preparation (*yellow* highlight). Error bars represent standard error.

.

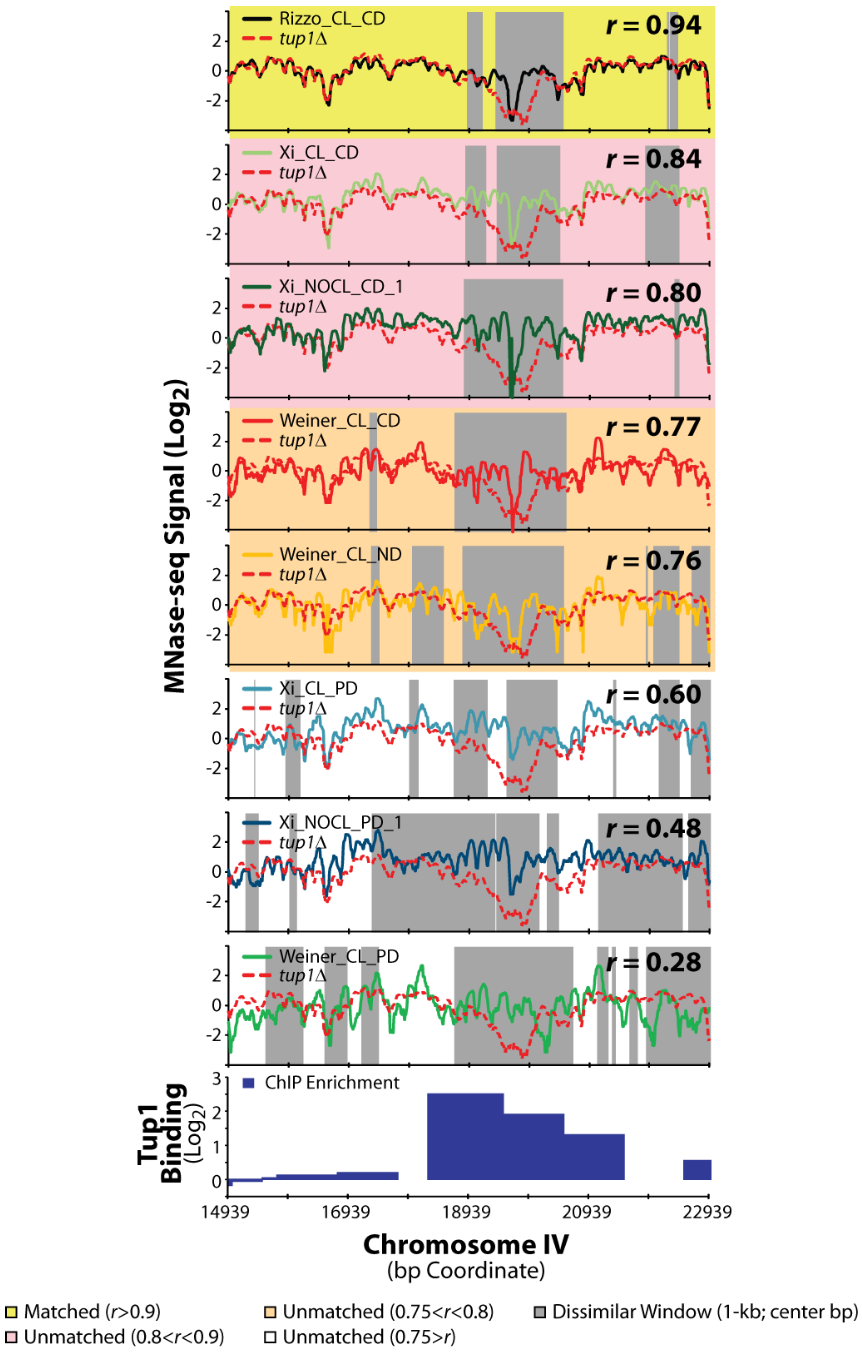

Supplemental Figure 3. Comparison between wild-type and *tup1*Δ MNase-seq experiments at a single region of Tup1-dependent chromatin

Graph illustrating MNase-seq signals, at 10-bp resolution, for a single region of chromatin. Graphs display data from our *tup1*Δ dataset (*red*) compared to various wild-type datasets. Sources and characteristics of wild-type datasets are listed in **Supplemental Table 1**. Graphs of dataset comparisons are sorted by genome-to-genome Pearson correlations between processed wild-type and *tup1*Δ MNase-seq datasets, whereby correlation (*r*) decreases reading from the top graph (wt = Rizzo_CL_CD) to bottom (wt = Weiner_CL_PD). *Gray bars* reflect windows of dissimilarity between chromatin structures, whereby the correlation between the two MNase-seq datasets is *r*<0.5 for a 1000 bp window centered on that base-pair. A bar graph of Tup1 binding by ChIP-chip analysis (*blue*; Bottom Graph) is also displayed for this same region. Notice how the number of dissimilar chromatin regions identified in MNase-seq datasets comparisons is inversely proportional to the Pearson correlation between datasets. Dissimilar chromatin structures between our matched experiments (*yellow* highlight) and other highly correlated datasets (*pink* and *orange* highlights) center on Tup1 binding events, whereas dissimilarities between unmatched chromatin structures do not, due to higher levels of technical variance. Identifications of non-specific changes in chromatin structure occur more frequently in poorly correlated and unmatched sample comparisons.

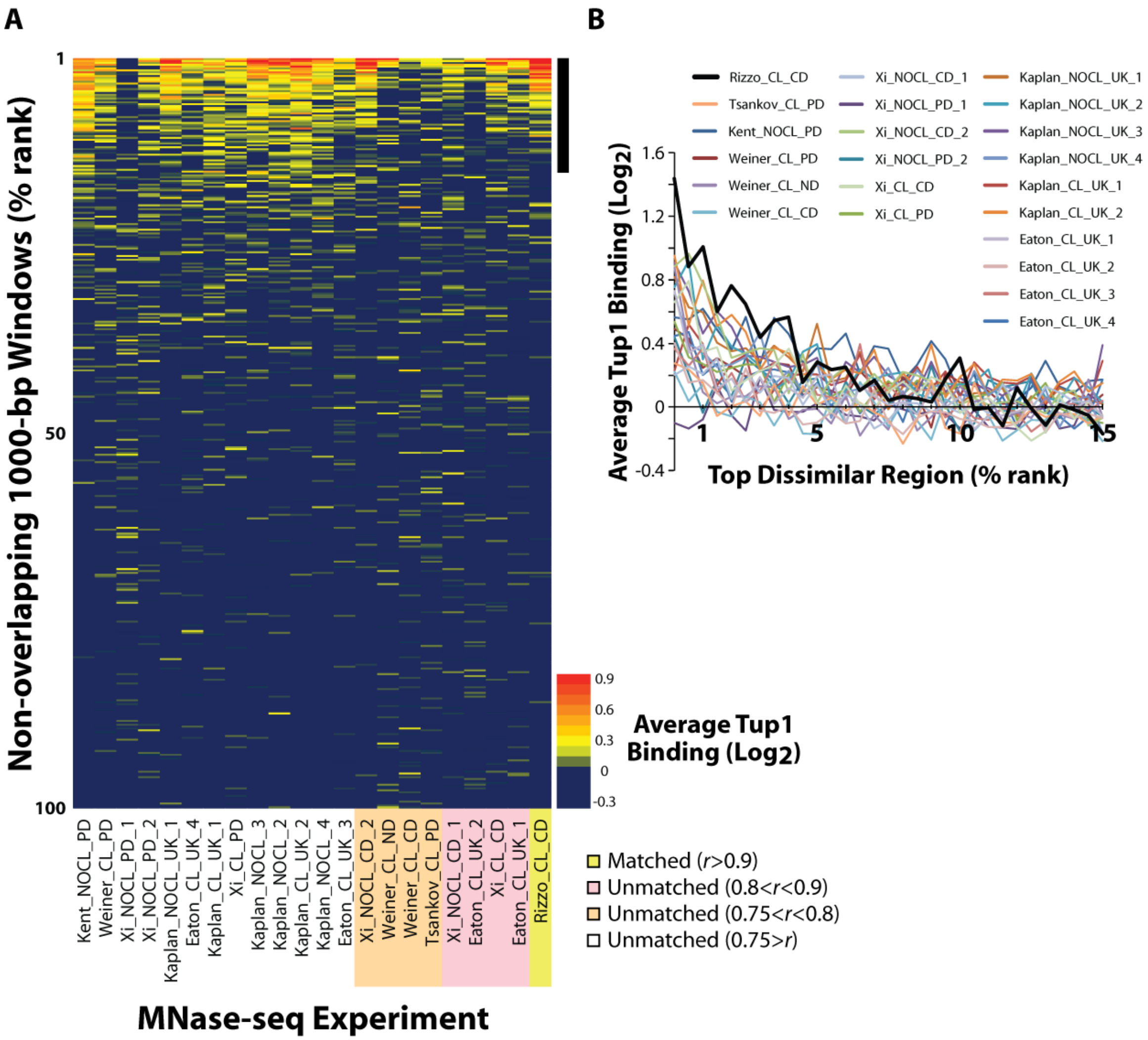

Supplemental Figure 4. The ability of matched MNase digests to specifically detect biologically-relevant differences in chromatin structure is NOT dependent on dissimilarity cutoff values used in analysis.

Results illustrate how the improved biological-signal-to-technical-noise ratio seen for our matched MNase-seq datasets is not contingent on the dissimilarity cutoff (r <0.5) used in the initial analysis.

A.) Heatmap illustrating the average Tup1 enrichment for all unique 1000 bp chromatin windows sorted by the percent rank of the Pearson correlation coefficient (r) from comparisons between *tup1*Δ MNase-seq data and various wild-type MNase-seq datasets (*x-axis*). **Supplemental Table 1** provides the experimental characteristics of MNase-seq datasets used in this analysis. Only chromatin regions with available MNase-seq and ChIP-chip Tup1 binding data [2] were used in this analysis and the average Tup1 binding profiles for this data was calculated for each 0.25% percentile of data. Our matched dataset (*yellow highlight*) shows an enrichment of Tup1 binding that is specific to the top ranked chromatin widows (i.e. most dissimilar regions between wild-type and *tup1*Δ datasets). Conversely, unmatched dataset comparisons show a marked increase in non-specific Tup1 binding signals at regions that are not strongly dissimilar chromatin regions. *Black bar* notes the most dissimilar chromatin regions (top 15% rank) identified in this analysis and used to populate the line graph in *SF 4B*.

B.) Line graph illustrating the average Tup1 binding data for the strongest dissimilar chromatin regions identified by analysis in *SF 4A*. Our matched dataset (*black*) consistently shows a stronger Tup1-enrichment score for the most dissimilar regions of chromatin identified.

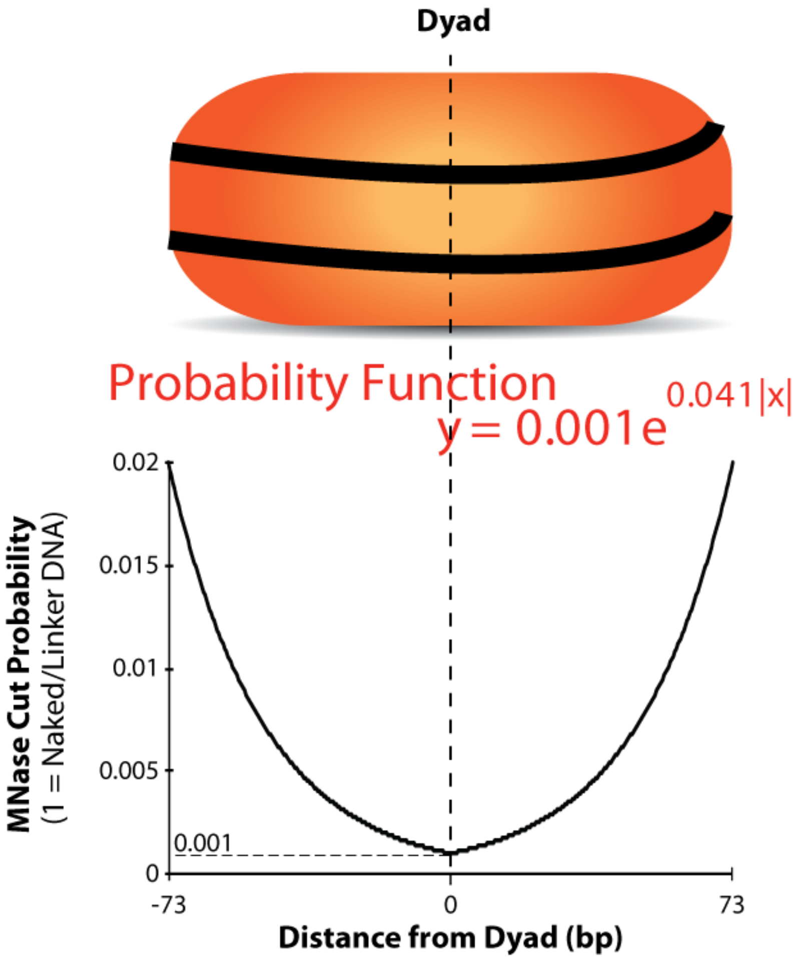

Supplemental Figure 5. MNase cut probability function for nucleosomal DNA templates.

The probability of cutting nucleosomal DNA templates during MNase digestion simulations was weighted using an exponential decay function (*red*) that relates MNase protection to a base-pair's location within a nucleosome. Protection was set to be 1000X greater than naked (linker) DNA at the nucleosome dyad with a decay to 50X protection at the edges of nucleosomes, based on the *in vivo* work of Widom and colleagues [3].

UNID	Strain	Growth Conditions	Formaldehyde Treatment	MNase Treatment (extent digestion)	Reference	SRA Run #	Correlation to *tup1*Δ (*r*)
Eaton_CL_UK_1	W303	Async; 23C	crosslinked	NA		SRR034470	0.8675993
Eaton_CL_UK_2	W303	Sync (G1); 37C	crosslinked	NA		SRR034477	0.806905165
Eaton_CL_UK_3	W303	Sync (G2); 37C	crosslinked	NA		SRR034471	0.723975647
Eaton_CL_UK_4	W303	Sync (G2); 37C	crosslinked	NA	[4]	SRR034472	0.563212133
Kaplan_CL_UK_1	BY4741	Async; 30C	crosslinked	NA		SRR023800	0.568898151
Kaplan_CL_UK_2	BY4741	Async; 30C	crosslinked	NA		SRR023801	0.669833108
Kaplan_NOCL_UK_1	BY4741	Async; 30C	native	NA		SRR023802	0.550596711
Kaplan_NOCL_UK_2	BY4741	Async; 30C	native	NA		SRR023803	0.66395226
Kaplan_NOCL_UK_3	BY4741	Async; 30C	native	NA		SRR023804	0.652363572
Kaplan_NOCL_UK_4	BY4741	Async; 30C	native	NA	[5]	SRR023805	0.687102203
Kent_NOCL_PD	BY4742	Async; 29C	native	Partial	[6]	SRR058444**	0.224414901
Rizzo_CL_CD	BY4741	Async; 30C	crosslinked	Complete		SRR353537	0.937185406
*tup1*Δ	BY4741/*tup1*Δ	Async; 30C	crosslinked	Complete	[1]	SRR353538	1
Tsankov_CL_PD	BY4741	Async; 30C	crosslinked	Partial	[7]	SRR059727	0.78024642
Weiner_CL_CD	BY4741	Async; 28C	crosslinked	Complete		SRR032451	0.774040861
Weiner_CL_ND	BY4741	Async; 28C	crosslinked	Normal		SRR032450	0.763686701
Weiner_CL_PD	BY4741	Async; 28C	crosslinked	Partial	[8]	NA*	0.277799503
Xi_CL_CD	JKM139	Async; 28C	crosslinked	Complete		SRR090259	0.837132088
Xi_CL_PD	JKM139	Async; 28C	crosslinked	Partial		SRR090258	0.602718195
Xi_NOCL_CD_1	JKM139	Async; 28C	native	Complete		SRR090251	0.801048411
Xi_NOCL_CD_2	JKM139	Async; 28C	native	Complete		SRR090253	0.759348555
Xi_NOCL_PD_1	JKM139	Async; 28C	native	Partial		SRR191761	0.484025371
Xi_NOCL_PD_2	JKM139	Async; 28C	native	Partial	[9]	SRR191762	0.548988243

*Dataset acquired directly from the author ; **Paired-end run

Supplemental Table 1: MNase-seq datasets used in this study

Sources and characteristics of technical preparation of the MNase –seq datasets used in this analysis. A range of different chromatin preparations, including variable crosslinking, growth, temperature and MNase-digestion extents, were included in this analysis to intentionally introduce technical differences into MNase-dataset comparisons and gauge their effect on signal comparisons. All experiments used chromatin from either wild-type (*black*) or *tup1*Δ (*red*) *S. cerevisiae* strains. All samples were grown in rich medium (YPD) to log phase and sequenced on the Illumina platform. All data were downloaded from the Sequence Read Archive Database (SRA) and processed identically, as described in *Methods* [10]. UNID = unique identifier, PD = Partial Digestion (<70% Monos), ND = Normal Digestion (70-89% Monos), CD = Complete Digestion (90-100% Monos), UK = Unknown Digestion (not specified). Correlation to *tup1*Δ = genome-to-genome Pearson correlation (*r*) between processed wild-type and tup1Δ MNase-seq datasets. Wild-type MNase-seq datasets represent a range of chromatin preparations, including our matched wild-type dataset which had the highest correlation to *tup1*Δ chromatin ($r = 0.93$). Other unmatched datasets represented a range of correlations to our *tup1*Δ data including well-correlated datasets ($0.75 < r < .9$) and poorly correlated datasets ($r < 0.75$).

References

1. Rizzo JM, Mieczkowski PA, Buck MJ: **Tup1 stabilizes promoter nucleosome positioning and occupancy at transcriptionally plastic genes**. *Nucleic Acids Res* 2011.
2. Hanlon SE, Rizzo JM, Tatomer DC, Lieb JD, Buck MJ: **The stress response factors Yap6, Cin5, Phd1, and Skn7 direct targeting of the conserved co-repressor Tup1-Ssn6 in S. cerevisiae**. *PLoS One* 2011, **6**(4):e19060.
3. Polach KJ, Widom J: **Mechanism of protein access to specific DNA sequences in chromatin: a dynamic equilibrium model for gene regulation**. *J Mol Biol* 1995, **254**(2):130-149.
4. Eaton ML, Galani K, Kang S, Bell SP, MacAlpine DM: **Conserved nucleosome positioning defines replication origins**. *Genes Dev* 2010, **24**(8):748-753.
5. Kaplan N, Moore IK, Fondufe-Mittendorf Y, Gossett AJ, Tillo D, Field Y, LeProust EM, Hughes TR, Lieb JD, Widom J *et al*: **The DNA-encoded nucleosome organization of a eukaryotic genome**. *Nature* 2009, **458**(7236):362-366.
6. Kent NA, Adams S, Moorhouse A, Paszkiewicz K: **Chromatin particle spectrum analysis: a method for comparative chromatin structure analysis using paired-end mode next-generation DNA sequencing**. *Nucleic Acids Res* 2011, **39**(5):e26.
7. Tsankov AM, Thompson DA, Socha A, Regev A, Rando OJ: **The role of nucleosome positioning in the evolution of gene regulation**. *PLoS Biol* 2010, **8**(7):e1000414.
8. Weiner A, Hughes A, Yassour M, Rando OJ, Friedman N: **High-resolution nucleosome mapping reveals transcription-dependent promoter packaging**. *Genome Res* 2010, **20**(1):90-100.
9. Xi Y, Yao J, Chen R, Li W, He X: **Nucleosome fragility reveals novel functional states of chromatin and poises genes for activation**. *Genome Res* 2011, **21**(5):718-724.
10. Leinonen R, Sugawara H, Shumway M: **The sequence read archive**. *Nucleic Acids Res* 2011, **39**(Database issue):D19-21.
