# Supplementary material for: Standardized collection of MNase-seq experiments enables unbiased dataset comparisons
Source: BMC Mol Biol. 2012 May 6;13:15. doi: 10.1186/1471-2199-13-15 (PMC3464627; doi:10.1186/1471-2199-13-15)
Supplement: Additional file 2 — Supplemental Figure 1. Unmatched preparations show poor consistency in identifying changes in MNase protection signals. Supplemental Figure 2. Matched MNase digests identify biologically relevant differences in chromatin structure. Supplemental Figure 3. Comparison between wild-type and tup1? MNase-seq experiments at a single region of Tup1-dependent chromatin. Supplemental Figure 4. The ability of matched MNase digests to specifically detect biologically-relevant differences in chromatin structure is NOT dependent on dissimilarity cutoff values used in analysis. Supplemental Figure 5. MNase cut probability function for nucleosomal DNA templates. Supplemental Table 1: MNase-seq datasets used in this study. [file 1471-2199-13-15-S2.pdf]

Supplemental Methods: Standardized MNase-seq Analysis

Alignment of unique (mappable) short-read sequences

All MNase-seq sequence reads should be aligned to their respective reference genome using the same alignment algorithm, parameters for alignment (number of mismatches allowed), and genome-build aligned to. Using this alignment algorithm, unique (mappable) regions of the genome should be identified and separated from non-unique (un-mappable) sequences. The latter should be removed from downstream analysis and should be identified in a reader-accessible database.

Extrapolating nucleosomal signals from aligned single-end sequences

Chromatin researchers often elect to process sequencing data by artificially extending short single-read sequences to assumed nucleosomal lengths (147 bp), to view nucleosome positioning and occupancy data in a more biologically relevant context. Uniform extension to 147 bp assumes that sequenced nucleosomal DNA populations are homogenous in size, which may not be accurate given differences in MNase digestion sequence biases, experimental sequence-specific biases and variability in the length of the measured nucleosomal DNA sequences[1-5]. Despite these caveats, uniform extension is still the most widely used analytical technique in the literature and can be a valuable tool for MNase-seq analysis if employed properly. Optimized uniform extension lengths can be selected by identifying the maximum correlation between forward and reverse sequencing tags [6]. This approach assures avoidance of over-extending DNA templates. The need to avoid over-extension is not surprising, because whenever single-end data are extended the extended sequences are *predicted*. Therefore, there are some inaccuracies with extended data, and the likelihood of introducing these inaccuracies increases with the length of the extension. For example, with a 36-bp single-end read we can be 100% sure that 37 bp were sequenced or even 100 bp, given the size selection of DNA material for sequence library preparations. However, we cannot be 100% sure that 147 bp were sequenced, and the further the data are extended the more likely that inaccuracies will be introduced into downstream analyses.

After extension of reads, each dataset needs to be standardized to correct for differences in the number of sequencing reads in each experiment (**Eq. 1**). Additional standardization measures include converting data into a relative ratio, which corresponds to deviation from average (**Eq. 2**). Many researchers elect to further transform this relative ratio into log-space by taking the log_2 [7-8]. This transformation is popular because it moves the data from ratio-space into continuous-space, thus enabling the use of standard statistical techniques. The added challenge with converting data into $\log_2$ ratios is that sites with zero sequence reads will be undefined. Therefore, the lowest theoretical value can be substituted at these locations to enable calculation (**Eq. 3**). Following standardization, MNase-seq datasets may be grouped together or viewed individually for conventional signal normalization (e.g. Z-score statistic) if warranted.

$$(\boldsymbol{Eq\ 1})\quad \#\, reads\ at\ bp_{(std\ 1\ mil)} = \frac{1 \times 10^6}{total\ \#\ mapped\ reads} \times \#\ of\ extended\ reads\ at\ bp$$

Eq. 1: **Standardization of sequence read counts to 1 million reads**. Where *"total # of mapped reads"* is the total of number sequence reads which align uniquely to the genome and "*# of extended reads at bp"* is the number of extended mapped reads covering that base pair.

$$(\boldsymbol{Eq}\ \mathbf{2})\ \ MNase\ protection\ = \frac{\#\ reads\ at\ bp_{(std\ 1\ mil)}}{AVERAGE(\#\ reads\ at\ bp_{(std\ 1\ mil)})}$$

Eq. 2: **Transforming standardized data to a relative ratio**. Where "*AVERAGE(# reads at bp$_{(std\ 1\ mil)}$)*" is the average number of reads for all base pairs in the genome.

$$(\boldsymbol{Eq}\ \mathbf{3})\ \ MNase\ protection_{(at\ 0\ site)}\ = \frac{1 \times 10^6}{(total\ \#\ mapped\ reads + 1) \times\ AVERAGE(\#\ reads\ at\ bp_{(std\ 1\ mil)})}$$

Eq. 3: **Determining the lowest theoretical value.** A single read is added to total # mapped reads for sites with zero mapped reads, thus defining the value if one additional read was sequenced and it was at this location.

In addition to uniform extension, alternative approaches to the analysis of MNase-seq datasets include the use of a nucleosome prediction algorithm (template filtering) developed by Weiner *et al* [5]. This program examines the distribution of single-end sequencing reads for MNase-seq experiments and fits this data to optimize the extension lengths of individual sequenced nucleosomal DNA templates, enabling variable extension sizes ranging from 80 to 220 bp. Template filtering outputs a set of predicted nucleosomes positions and occupancies. Use of variable extension in the analysis of MNase-seq datasets removes assumptions of uniform extension. Despite this advantage, however, the identification of individual nucleosome DNA templates by template filtering makes alternative assumptions regarding called nucleosome template overlaps and relative abundances and may eliminate sequences from analysis to optimize the fit of data to specific chromatin structures. In contrast, uniform extension of single read MNase-seq datasets incorporates all sequencing data into the analysis and may be of additional value in investigating low-abundance variations in chromatin.

Identifying differences between MNase-seq datasets

To identify sites of dissimilarly between two datasets we suggest using both extended data and nucleosome predictions (template filtering). When comparing nucleosome predictions, one can search between datasets for different called nucleosome locations and occupancies. The significance of changes in occupancy and/or location for called templates between two datasets can be tested using non-parametric approaches. Changes of interest can be compared to changes seen from a random sampling of predicted nucleosomes from both datasets when sampling the remainder of the genome [9].

Alternatively, comparisons between extended $\log_2$ ratio datasets can be done by sliding a window across both datasets and calculating a Pearson correlation. Windows with a low correlation can identify regions of dissimilarity between the two MNase-seq datasets. Both the size and correlation cutoffs for dissimilar windows can be adjusted to allow for more lenient definitions of dissimilar chromatin structures if/when necessary. Cutoff adjustments may be guided by examining the histogram distribution for all correlation windows (**Figure 4**).

References

1. Dingwall C, Lomonossoff GP, Laskey RA: **High sequence specificity of micrococcal nuclease**. *Nucleic Acids Res* 1981, **9**(12):2659-2673.
2. Horz W, Altenburger W: **Sequence specific cleavage of DNA by micrococcal nuclease**. *Nucleic Acids Res* 1981, **9**(12):2643-2658.
3. Kaplan N, Hughes TR, Lieb JD, Widom J, Segal E: **Contribution of histone sequence preferences to nucleosome organization: proposed definitions and methodology**. *Genome Biol* 2010, **11**(11):140.
4. Floer M, Wang X, Prabhu V, Berrozpe G, Narayan S, Spagna D, Alvarez D, Kendall J, Krasnitz A, Stepansky A *et al*: **A RSC/nucleosome complex determines chromatin architecture and facilitates activator binding**. *Cell* 2010, **141**(3):407-418.
5. Weiner A, Hughes A, Yassour M, Rando OJ, Friedman N: **High-resolution nucleosome mapping reveals transcription-dependent promoter packaging**. *Genome Res* 2010, **20**(1):90-100.
6. Lai WK, Bard JE, Buck MJ: **ArchTEx: accurate extraction and visualization of Next-Generation Sequence data**. *Bioinformatics* 2012.
7. Lee W, Tillo D, Bray N, Morse RH, Davis RW, Hughes TR, Nislow C: **A high-resolution atlas of nucleosome occupancy in yeast**. *Nat Genet* 2007, **39**(10):1235-1244.
8. Kaplan N, Moore IK, Fondufe-Mittendorf Y, Gossett AJ, Tillo D, Field Y, LeProust EM, Hughes TR, Lieb JD, Widom J *et al*: **The DNA-encoded nucleosome organization of a eukaryotic genome**. *Nature* 2009, **458**(7236):362-366.
9. Rizzo JM, Mieczkowski PA, Buck MJ: **Tup1 stabilizes promoter nucleosome positioning and occupancy at transcriptionally plastic genes**. *Nucleic Acids Res* 2011.
