# Supplementary material for: Standardized collection of MNase-seq experiments enables unbiased dataset comparisons
Source: BMC Mol Biol. 2012 May 6;13:15. doi: 10.1186/1471-2199-13-15 (PMC3464627; doi:10.1186/1471-2199-13-15)
Supplement: Additional file 3 — Supplemental Methods: Standardized MNase-seq Analysis. [file 1471-2199-13-15-S3.pdf]

Buck Lab Protocols

Matched Micrococcal Nuclease Digestions

Source: Jason Rizzo and Michael Buck

Schematic of Protocol Work-Flow

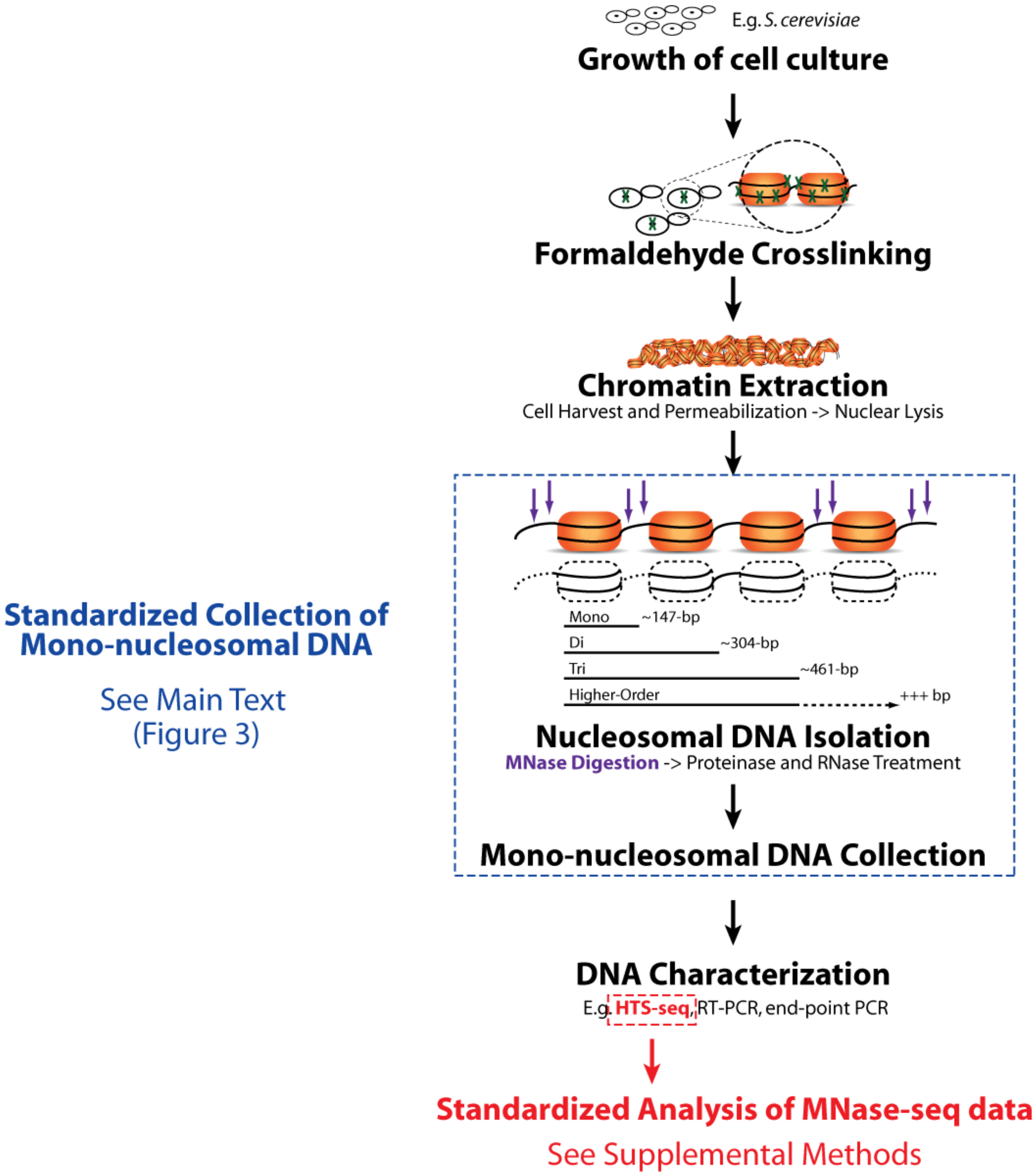

Note: HTS-seq = High-throughput sequencing

Matched Micrococcal Nuclease Digestions

Source: Jason Rizzo and Michael Buck

<u>Materials</u>

Reagents

37% Formaldehyde (Fisher; Cat. no: F79-1)

> ***CAUTION*** *Formaldehyde is flammable; highly toxic by inhalation, contact with skin or if swallowed; causes burns; and is potentially carcinogenic. Formaldehyde should be used with appropriate safety measures, such as protective gloves, glasses and clothing, and adequate ventilation. Formaldehyde waste should be disposed of according to regulations for hazardous waste.*

2.5 M Glycine (Sigma; Cat. no: G8790)

10X Phosphate buffered saline (PBS) (Invitrogen/Gibco; Cat. no.:14200-075)

1X PBS (diluted from 10X PBS)

95% ethanol (Aaper; Cat. No.:111000190)

Dry-ice

Coomassie Plus (Bradford) Protein Assay (Thermo Scientific; Cat. no.: 1856210)

Sodium Chloride (NaCl) (Fisher; Cat. no.: S671)

Glycerol (Fisher; Cat. no.: G31)

EDTA (Fisher; Cat. no.: BP2927)

1M Tris-HCl pH 7.5 & 8.0 (Cat. no.: BP1757 & BP1758)

Calcium chloride dehydrate (CaCl2) (Fisher; Cat. No.: BP510)

Micrococcal Nuclease (MNase) (Worthington Biochemical Corp; Cat. no.: LS004797)

> ***NOTE****: Stock MNase is typically 10U/µl; Stored at -80C in 10-15µl aliquots in MNase Storage Buffer (see Reagent Setup)*

Sodium Dodecyl Sulfate (SDS) (Fisher; Cat. No.: BP166)

20 mg/ml Proteinase K solution (Invitrogen; Cat. no.: 25530-049)

3M Sodium acetate, pH 5.2 (Sigma; Cat. no.: S-7899)

Phenol:chloroform;premixed with isoamyl alcohol (Amresco; Cat. no.: 0883)

> ***CRITICAL****: Buffer to pH 8.0; If this solution is old or is at low pH, there will be degradation of DNA.*
>
> ***CAUTION:*** *Phenol is toxic when in contact with skin or if swallowed; causes burns; and is irritating to eyes, the respiratory system and skin. Chloroform is harmful by inhalation or if swallowed; is irritating to skin; and is potentially carcinogenic. Isoamyl alcohol is flammable; and is irritating to eyes, the respiratory system and skin. The phenol:chloroform:isoamyl alcohol should be used with appropriate safety measures, such as protective gloves, glasses and clothing, and adequate ventilation.The phenol:chloroform:isoamyl alcohol waste should be disposed of according to regulations for hazardous waste.*

Matched Micrococcal Nuclease Digestions

Source: Jason Rizzo and Michael Buck

> ***NOTE:*** *Standard liquid organic solvent extractions can be replaced with Phase lock, heavy, pre-dispensed into 2.0 ml microfuge tubes (Eppendorf)*

70% ethanol (diluted from 95% ethanol)

1X TE Buffer, pH 8.0 (Amresco; Cat. no.: E112)

10 mg/ml RNAseA (Sigma; Cat. no.: R6513)

Agarose (Multi-Purpose) (Fisher; Cat. No.: BP160)

Xylene Cyanol (Fisher; Cat. No.: O6116)

Ficoll (Acros organics; Cat. No.: AC61186-0050_

Double-distilled water

For making and diluting solutions

Nuclease-free water

For diluting reactions and smaller volumes (x < 1ml)

Matched Micrococcal Nuclease Digestions

Source: Jason Rizzo and Michael Buck

Reagent Setup

Nuclease Digestion Buffer (DB)
10 mM Tris-HCl pH 8.0
1mM $CaCl_2$

Stop Solution
8.6% SDS
0.007 M EDTA
For 110μl add 95μl 10% SDS + 15μl 0.5M EDTA (pH 8)

MNase Storage Buffer
10mM Tris-HCl, pH 7.4
50mM NaCl
1mM EDTA
50% Glycerol

10X DNA Loading Buffer (without Bromphenol Blue)
20% Ficoll
0.1 M EDTA
1% SDS
0.25% Xylene Cyanol
For 10ml (0.2g Ficoll; 2 ml 0.5M EDTA stock; 1 ml 10% SDS Stock; 0.025g xylene cyanol; fill upto 10ml with ddH2O).

Matched Micrococcal Nuclease Digestions

Source: Jason Rizzo and Michael Buck

Equipment

Mini-beadbeater-8 and 0.5mm glass beads (Biospec Products, Inc.)

Standard spectrophotometer (Biorad); Capable of reading OD_{600} and OD_{595})

Swinging bucket centrifuge for 50ml conicals, variable temperature (e.g., Sorvall Legend RT)

High-speed table-top centrifuge for 1.5ml samples (>13,000rpm; variable temperature)

65C degree water bath

18G, ½ inch needles (BD; Product Number: 305196)

Buck Lab Protocols

Matched Micrococcal Nuclease Digestions

Source: Jason Rizzo and Michael Buck

Protocol

Growth of cell culture

1. Grow cells under the desired conditions and to the desired density.

   S. cerevisiae: Want 200ml at ~1 OD_{600}.

   Protocol can be scaled up proportionately.

Formaldehyde crosslinking

2. Add formaldehyde directly to the culture, to a final concentration of 1%. Incubation for 15-30min at room temp and 200rpm.

   For a 200 ml culture, this is 5.4 ml of a 37% formaldehyde stock.

3. Stop crosslinking with Glycine (from a 2.5 M stock) to a final concentration of 125 mM. Incubate for 5 minutes at room temperature (quenches reaction).

   For a 200 ml culture, 10 ml 2.5 M Glycine.

4. Pellet the cells in 50 mL conical tubes at 1500 rpm for 5 mins.

5. Wash pellet twice with 50 mL 1X PBS. Vortex and centrifuge at 1500rpm for 5mins after each wash and then quick freeze the pellet with liquid nitrogen or dry-ice and EtOH. Store at -80C until ready to continue.

   Pause-point. *Once cells are crosslinked, they may be stored frozen at −80 °C indefinitely.*

Chromatin extraction (Mechanical)

6. Thaw sample on ice and resupend into 1 ml DB buffer and transfer to 1.5 ml tube. Pellet cells, full speed 5 mins and aspirate all liquid.

   Troubleshooting. *It is important to remove all liquid so that the pelleted cells can be weighed accurately.*

   Critical step*: From here on keep pellets and future lysates on ice*

7. Weigh cells, ensuring that you blank with empty tube.

8. Re-suspend pellets weighing less than 0.2 mg with 400μl of DB buffer. If your pellet weighs more than 0.2 mg then split into 400μl samples with less than 0.2 mg of cells. Make sure the cells are completely resupended before continuing.

9. Add glass beads ~1ml of 0.5mm glass beads. Fill up to the 1.5 ml mark on the tube.

   Troubleshooting. *Make sure that none of the glass beads are around the lip when closing the tube. They can disrupt the seal causing the tube to leak in the beadbeater.*

10. Lyse cells in a mini-beadbeater-8 (Biospec) with four one-minute sessions at the highest setting. Place tubes on ice for two minutes between each session.

11. Recover the extract by punching a small hole in the bottom of the tube with a 18G, ½ inch needle. Spin the liquid through to another tube with a one-pulse-spin for 30 seconds. Repeat 2-3X to clear lysate from tube.

__Critical step__: Make sure centrifuge is adequately balanced. Do NOT exceed 2,500 rpm.

12. Combine all samples from the same culture together and determine the protein concentration by a Bradford assay (OD_{595}).

For *S. cerevisiae*, protein yields should be between 4-22 mg total protein for a 400µl sample (~36-80 OD_{595} Units).

__Critical step__: Ensure consistent yields between chromatin preparations you intend on comparing

13. Quick-freeze the samples with liquid nitrogen or a (dry-ice + 95% ethanol) bath.

Pause-point. *Lysates may be stored frozen at −80 °C indefinitely.*

__Critical step__: Samples should be stored in aliquots to limit freeze thawing (5.5 mg per aliquot allows 5 MNase digestions). Even if you plan to continue with digestions on the same day, all samples should still be frozen so that if you need to return to the other aliquots results will be reproducible.

Nucleosomal DNA Isolation

Micrococcal nuclease (MNase) digestion

14. MNase digestions should be performed on **1 mg of total protein in 200*µl* DB** for **1 hour at 37 C**. *Digest titration:* Three to five test digestions should be setup with a range of total units added (for *S. cerevisiae*: 50, 25, 10, 5, and 1 units total added). This titration helps to identify the amount of MNase needed to achieve your desired extent of digestion. This result will be specific to each chromatin preparation and MNase stock used.

We recommend collection of complete-digested samples for most consistent chromatin collection/sampling (see Manuscript).

Note: Additional digests across a larger range of concentration may be necessary for other cell types

__Critical step__ Always store MNase at -80 in 10-15µl aliquots. For consistency/reproducibility: thaw each aliquot once only; discard all un-used..

__Critical step:__ When setting up MNase digests, first mix all thawed aliquots needed together, then prepare a diluted MNase stock for lower concentration digests (e.g. dil stock = 5µl stock + 45µl DB; use 5µl dil stock for 5U/200µl reaction;).

__Critical step:__ Never pipette less than 1µl MNase (use dilutions accordingly).

__Critical step__: Use a 37C water bath to ensure even heating.

15. Stop reaction with 29*µl* stop solution, make this fresh.

Vortex samples upon stop solution addition.

Matched Micrococcal Nuclease Digestions

Source: Jason Rizzo and Michael Buck

***Critical step**: Longer digestion times (1 hour) make results less time sensitive. However, if collecting a large number of samples, stagger preparation of MNase digests to ensure similar start-stop digest times.*

Proteinase/RNase treatment and DNA precipitation

16. Add 2μl Proteinase K (20 mg/ml). Incubate 30min at room temperate and at 65C for 6 hr to overnight.

***Critical step**: Use a 65C water bath to ensure even heating. Be sure to check water levels for evaporation.*

17. Add 300 μl Phenol:Chloroform:Isoamyle alcohol in each tube. Vortex and spin for 4 min at 14,000rpm and 4C. Transfer aqueous (top) layer to a new tube. Add addition 100μl DB buffer to PCI, vortex and spin and transfer aqueous combining with pervious.

18. To the aqueous layer extract again with 300 μl Phenol:Chloroform:Isoamyle alcohol, vortex, spin and transfer aqueous layer to new tube.

19. Add 30 μl NaAcetate 3M pH 5.2, mix.

20. Ethanol precipitate with 1.3 ml 95%, mix. Spin down at 4C for 20 min, at 14,000 rpm and dump out the ethanol.

***Pause-point.** DNA precipirates may be stored in 95% EtOH at −80 °C indefinitely.*

21. Rinse pellet with 70% ethanol. Spin down for 10 min, at 14,000 rpm and dump again out the ethanol. Try to remove as much ethanol as possible to allow easy drying.

22. Dry pellet by placing open tube upside down in the hood for 30 mins, or spinning in speed vac for 5 mins.

***Critical step**: Avoid using a heated vacuum to minimize sample degradation.*

23. Resuspend in 50 μl TE + 2 μl RNase (10 mg/ml) and incubate at 37C for 30 min.

***Critical step**: Use a 37C water bath to ensure even heating.*

***Pause-point.** DNA may be stored at −20 °C for 3-6 months.*

Mono-nucleosomal DNA collection

24. Run 10μl of each sample with 2μl 10X loading buffer on 2% agarose gel. Select sample which is complete-digested, has only a single tight band at 120-150 bps. If there are larger bands then the sample is not digested enough. The remaining 40μl can be purified directly using a column (E.g. Zymo Research).

Typical yield should be 600-800 ng DNA per 1mg digested WCE. Additional samples may not be needed depending on downstream applications.

***Critical step**. Use a DNA loading buffer that does not include Bromophenol Blue dye, which runs around 300bp and may interfere with image analysis. See: Reagent Setup*

***Troubleshooting.** Sample concentrations (for downstream characterizations) are best determined using picogreen.*

Repeated mono-nucleosomal DNA collections

25. Repeat Steps 14-24; Step 14: Conduct a focused titration (3-5 digests) using MNase concentrations both at and surrounding the previous replicate's ideal extent of digestion identified.

Selecting MATCHED MNase digestions

26. Run all of the chromatin DNA samples you wish to compare on the same 2% gel (10μl of each sample + 2μl *10X loading buffer* / gel lane) to identify matched digests using gel analysis (see next section).

***Critical step.** These samples should all be of a similar digestion extent for matching. We recommend complete-digest chromatin*

***Critical step**. Use a DNA loading buffer that does not include Bromophenol Blue dye, which runs around 300bp and may interfere with image analysis. See: Reagent Setup*

***Troubleshooting**. If comparing samples (e.g. wild-type vs. mutant), run paired samples on the same gel to collect a single matched replicate (e.g. wild-type matched to tup1Δ).*

27. Column purify MATCHED samples of interest for downstream characterization/analysis.

Troubleshooting. Many downstream protocols, such as high-throughput DNA sequencing, are extremely quantity-sensitive. Therefore, final concentrations of prepared mono-nucleosomal DNA are best determined using ultra-sensitive fluorescent nucleic acid staining techniques (i.e. PicoGreen dye), since this measure has a larger dynamic range and is specific for double-stranded DNA. Use of fluorescent quantification will ensure that you have sufficient dsDNA for downstream applications.

Matched Micrococcal Nuclease Digestions

Source: Jason Rizzo and Michael Buck

Gel Analysis:

Deciding on the correct digest conditions and pairing samples for comparison

From our qPCR analysis of selected sites it is clear that two biological samples can only be compared to each other if their digestions are exactly the same. In addition, complete digestion to only mono-nucleosome DNA ensures that all samples have the same population of fragments. We determine the correct digestion by running the sample on 2% gel for an extend time to make sure that you get good separation with 100-bp DNA ladder. When you take pictures with gel-image make sure that you save all images, and save different exposures. It will help if you make sure that your gels are straight when you take the pictures.

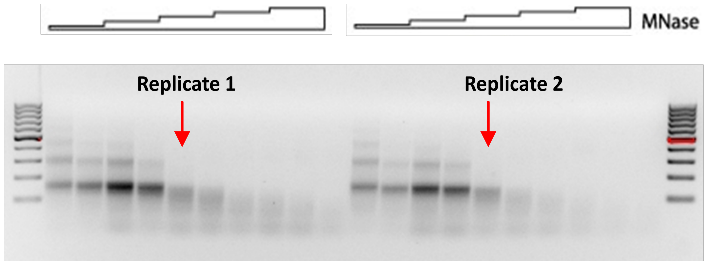

Protocol Figure 1. Samples should be digested until there is no detectable di-nucleosome band

Using gel image software to determine the density front of your samples.

1) Load images in Quantity one software.
2) Select Lane menu, and Frame Lanes. It will ask you for the number of lanes, and provide a frame of the lanes. Adjust the frame until the lanes are good.
3) Use the compare lane function to see the intensity between multiple lanes. It helps to select your ladder so that you can see the 100 and 200-bp bands. The images below are from this function. So should be able to judge from this images which are the best lanes.
4) Create a lane report – Select Reports – All lane report. Save a tab-delimited or excel format. This file will have intensities of each lane throughout the gel. We can use it to determine the correlation between two samples.
5) From the intensity profiles select the lanes with a single mono-nucleosome peak, without any bump at the di-nucleosomes (*red arrows* in **Protocol Figure 1**). This ensures that your samples are completely digested.
6) Run a second gel with all complete-digested chromatin preparations to identify digests that are extremely similar by determining the Pearson correlation between two smears (E.g. **Protocol Figure 2**). To compare two samples to each other the smears should be correlated $r > 0.9$ (See *Manuscript*: **Figure 5**).
7) Finish purifying the best pair of samples as described in **Step #27**. Run a final comparison gel for the two samples with 100 ng of DNA and ensure that correlation between the smears is $r > 0.9$. This can go directly into the Illumina sequence library protocol.

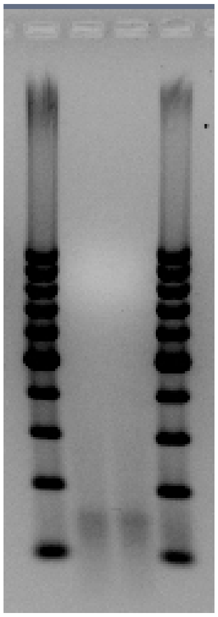

Protocol Figure 2. Paired samples for comparison. $r = 0.99$.
